# The mitochondrial-targeted peptide SBT-20 ameliorates inflammation and oxidative stress in chronic renal failure

**DOI:** 10.18632/aging.103681

**Published:** 2020-09-25

**Authors:** Lina Sun, Haiping Xu, Yunfei Wang, Xiaoying Ma, Yan Xu, Fuyun Sun

**Affiliations:** 1 Department of Nephrology, Cangzhou Central Hospital, Cangzhou, Hebei Province, China; 2 Department of Cardiology, Cangzhou Central Hospital, Cangzhou, Hebei Province, China

**Keywords:** chronic renal failure, mitochondrial-targeted peptide, SBT-20, oxidative stress, inflammation

## Abstract

Chronic renal failure (CRF) is the final outcome of the development of chronic kidney disease with different causes. Although CRF is a common clinical disease, its pathogenesis remains to be improved. SBT-20 belongs to a class of cell-permeable peptides that target the inner mitochondrial membrane, reduce reactive oxygen species (ROS), normalize electron transport chain function, and ATP generation. Our experiment was to evaluate whether SBT-20 affected the oxidative stress and inflammatory process of CRF. The levels of ROS production, mitochondrial membrane potential, NF- κB-p65, TNF-α, Drp1, and mfn2 were measured before and after SBT-20 treatment. We observed that SBT-20 treatment inhibited H2O2-induced mitochondrial ROS production. SBT-20 could also restore the mitochondrial membrane potential and reduce the elevated levels of NF-κB-p65 and TNF-α in HK-2 cells. *In vivo*, the renal function of CRF mice recovered after treating with SBT-20, the levels of necrotic cells and inflammation decreased, and the morphology of mitochondria recovered. The results showed that SBT-20 had a protective effect on CRF by reducing oxidative stress, inflammation progression *via* down-regulating of NF-κB-p65, TNF-α, and Drp1 and upregulating of Mfn2. These data support SBT-20 could be used as a potential preparation for CRF.

## INTRODUCTION

Chronic renal failure (CRF) refers to the decrease of glomerular filtration rate (glomerular filtration rate, GFR) and the irreversible damage of renal structure and function caused by various causes [1]. The renal parenchyma is seriously damaged, and renal function decreases slowly until failure. The clinical manifestations are decreased renal function, retention of metabolites, imbalance of electrolyte, and acid-base balance, and systemic symptoms [2]. It is inferred that hundreds of thousands of people suffer from CRF every year [3]. The incidence of chronic kidney disease in China has reached 10.8%, and it is still on the rise. Its prevention and treatment have become one of the important public health problems faced by countries all over the world. It is very import to explore the possible molecular mechanisms of CRF and identify new therapy.

Mitochondria play a key "switch" role in apoptosis [4]. Under the stimulation of apoptosis signal, the "life and death switch"-mitochondrial permeability transition pore was continuously opened, the outer membrane potential decreased, mitochondria swelled and ruptured, and apoptotic factors such as cytochrome C were released into the cytoplasm through cascade apoptotic protease activating factor-1 (Apaf-1) to activate caspase, making cells enter the apoptosis process [5]. Studies have shown that the classical mitochondrial pathway is involved in the apoptosis of renal tubular epithelial cells in rats with hyperoxaluria [6, 7]. It has been reported that the relationship between glomerulosclerosis and endothelial cell apoptosis in anti-GBM glomerulonephritis model showed that active necrosis disappeared after 4-8 weeks, but endothelial apoptosis increased significantly, which was consistent with the degree of glomerulosclerosis [8, 9].

In the past 20 years, chemists and biochemists have synthesized many structurally modified peptides to improve the ability to pass through the cell membrane and maintain low toxicity and immunogenicity. On this basis, polypeptides capable of targeting, such as mitochondrial targeting peptides, were developed [10]. SS-31 is a mitochondrial targeting peptide, which can clear mitochondrial ROS and inhibit MPT (mitochondrial phosphate transporter), suggesting that it can protect the ischemic renal injury. These results support MPT as an upstream target of drug intervention for IR damage and suggest that early protection of mitochondrial function can be used as a therapeutic strategy to prevent apoptosis and necrosis of renal tubular cells, reduce oxidative stress, and reduce inflammation [11]. SS-31 is expected to play an important role in the prevention and treatment of the acute renal injury. SS-31 can also reduce renal tubulointerstitial injury in diabetic mice by inhibiting mitochondrial division. The injectable KLDD nanofiber hydrogel can prolong the release of Mito-2,2,6,6-tetramethylpiperidine-N-oxyl *in vitro*, so as to provide continuous protection against IRI-induced renal mitochondrial dysfunction in mice, thus reducing renal tubular injury and inflammation after IRI [12]. This study suggests that SAP hydrogel is a promising therapeutic drug carrier to promote renal repair after renal injury. Such as MitoQ, Szeto-Schiller (SS) peptides, SkQ1, and SkQR1, and superoxide dismutase mimics were explored in heart diseases [13]. The favorable results obtained in the study of myocardial ischemia/ reperfusion injury also suggest strategies for the prevention of renal ischemia /reperfusion injury in the future.

Mitochondria-targeted peptide SBT-20, also known as SS-20 (Phe-D-Arg-Phe-Lys-NH2), is a cell-permeable, mitochondria-targeting tetrapeptide that belongs to the Szeto-Schiller (SS) peptides, which target the inner mitochondrial membrane, reduce ROS, normalize electron transport chain function and ATP generation. In a previous report, SBT-20 significantly reduced infarct size in myocardial injury [14]. The reduction of injury is considered to be related to the stability of mitochondrial function and the decrease of mitochondrial ROS production. We hypothesis that SBT-20 may improve the CRF by alleviating the mitochondrial function. Therefore, we aimed to detect the role of SBT-20 on CRF. In our research, HK-2 cells were added with SBT-20 after LPS /H2O2 treatment. SBT-20 alleviated the inflammation and oxidation in HK-2 injury *via* controlling the mitochondrial function. *In vivo*, SBT-20 prevented apoptosis and inflammation and recovered the renal function through restoring mitochondrial function.

## RESULTS

### The location and effect of SBT-20 in mitochondrial

**Figure 1:**
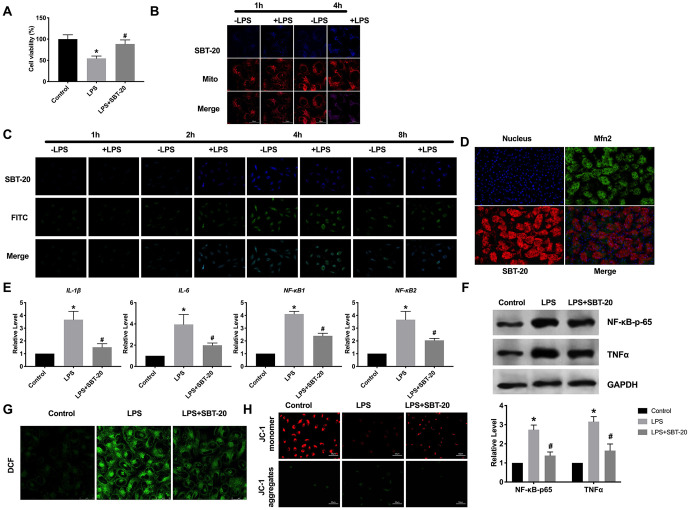
**The location and effect of SBT-20 in mitochondrial**. (**A**) MTT assay was carried out to assess the cell viability. n=10. (**B**) Representative diagram of cellular uptake of SBT-20. (**C**) Representative diagram of colocalization of SBT-20 with FITC. (**D**) Representative diagram of colocalization of SBT-20 with Mfn2. (**E**) The mRNA level of inflammatory-associated cytokine IL-1β, IL-6, NF-κB1 and NF-κB2 in different group. n=7. (**F**) The protein level of NF-κB-p65 and TNFα. (**G**) The ROS production was measured in cells. n=5. (**H**) Detection of mitochondrial membrane potential. (*P<0.05 *vs.* control group, ^#^*P*<0.05 *vs.* LPS group).

In order to evaluate the biocompatibility of SBT-20, the effect of SBT-20 on the cell viability of HK-2 was determined. We induced HK-2 cells with 400 ng/ml LPS, and then gave the cells 1 μM SBT-20, SBT-20 significantly restored the cell viability of LPS-treated cells close to the normal level (Figure 1A). Cellular uptake and mitochondrial uptake activity were investigated by SBT-20 with fluorescent tags. There was no significant difference in fluorescence intensity of normal HK-2 cells treated with SBT-20, indicating that SBT-20 had a good ability of cell uptake. The fluorescence intensity of SBT-20 in HK-2 cells stimulated by LPS was significantly higher than that in normal cells. In addition, the fluorescence of SBT-20 and Mito-Tracker overlap well in each group, indicating that SBT-20 has the ability of mitochondrial targeting. (Figure 1B). We also used double-labeled fluorescent nanoparticles (FITC-SBT-20) to detect the drug release ability of SBT-20 in HK-2 cells. The results showed that with the extension of time, the fluorescence of SBT-20 and FITC increased at first and then decreased, and reached the highest at 4 hours to 8 hours, indicating that SBT-20 was released from both normal and LPS-stimulated cells (Figure 1C). The mitochondrial fusion protein 2 (Mfn2) is located in the outer membrane of mitochondria and has the function of promoting mitochondrial fusion and maintaining the normal structure of mitochondria. Mfn2 exists widely in many organs and tissues of the whole body. Mfn2 promotes mitochondrial fusion and maintains the normal structure of mitochondria, participates in mitochondrial fusion, and affects mitochondrial metabolism. Fluorescence experiments show that there is a high degree of overlap between Mfn2 and SBT-20 (Figure 1D). In the model of inflammation induced by LPS, the level of inflammatory cytokines increased, SBT-20 treatment could prevent the expression level of the inflammatory-associated cytokine IL-1β, IL-6, NF-κB1 and NF-κB2 in LPS-treated cells (Figure 1E), which led to relief of inflammation. Western blot also performed similar results by detecting the level of NF-κB-p65 and TNFα (Figure 1F).

As a mitochondrial targeting peptide, there is no doubt that SBT-20 plays a role after entering into mitochondria, so the effect of SBT-20 on mitochondrial function needs to be further studied. Reactive oxygen species (ROS) is an inevitable product of cell metabolism, which can act on mitochondria and is one of the main ways to cause mitochondrial damage. Higher levels of ROS production were observed in the LPS-treated HK-2 cells. Interestingly, treatment with the SBT-20 reduced ROS production in the LPS-treated HK-2 cells (Figure 1G). The maintenance of mitochondrial membrane potential is a necessary condition for the realization of normal mitochondrial function. The decrease of mitochondrial membrane potential will lead to insufficient production of mitochondrial ATP, which in turn affects the normal life activity of cells, which is one of the earliest events in the process of apoptosis. JC-1 assay kit was used for detected mitochondrial membrane potential. JC-1 monomers were indicated as the mitochondrial depolarization. JC-1 aggregates were described as the normal mitochondrial membrane potential. The decreased mitochondrial membrane potential was recovered by SBT-20 treatment in LPS-treated HK-2 cells (Figure 1H).

### SBT-20 blocks oxidative stress induced by H_2_O_2_ in HK-2 cells

**Figure 2:**
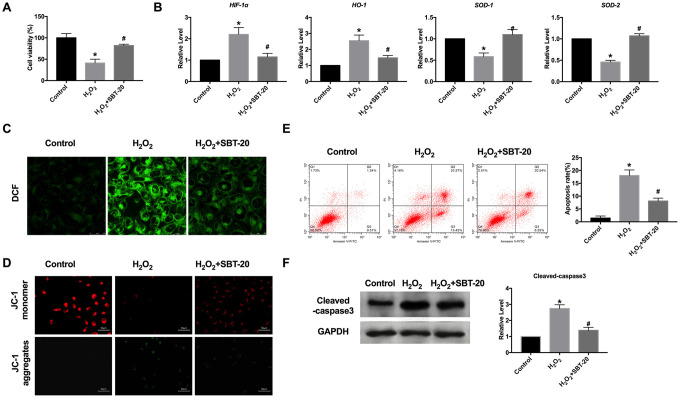
**SBT-20 blocks oxidative stress induced by H_2_O_2_ in HK-2 cells.** (**A**) MTT assay was carried out to assess the cell viability. n=10. (**B**) RT-PCR was employed for detecting oxidative stress-relative markers, HIF-1α, HO-1, SOD1 and SOD2. n=6. (**C**) The ROS production was measured in cells. (**D**) Detection of mitochondrial membrane potential. (**E**) The apoptotic cells was quantified using flow cytometry. n=3. (**F**) The protein level of cleaved-caspase3 was assessed. n=5. (*P<0.05 *vs.* control group, ^#^*P*<0.05 *vs.* H_2_O_2_ group).

Studies have found that the kidney is one of the organs highly sensitive to oxidative stress, oxidative stress throughout the development of nephropathy, the weakening of antioxidant capacity, and the enhancement of oxidative stress plays an important role in the occurrence and development of the renal disease. Oxidative stress can directly act on the polyunsaturated fatty acids of the renal cell membrane, cause lipid peroxidation, destroy the normal physiological state of the cell membrane, directly damage mitochondrial DNA, cause phospholipid peroxidation of the glomerular capillary basement membrane, and increase the permeability of glomerular basement membrane. We used 200 μM H2O2 to simulate the model of oxidative stress *in vitro*. MTT assay results showed that SBT-20 could save cell damage caused by H2O2 (Figure 2A). RT-PCR was employed for detecting oxidative stress-relative markers, including hypoxia-inducible factor 1α (HIF-1α), heme oxygenase-1 (HO-1), superoxide dismutase-1 (SOD1) and superoxide dismutase-2 (SOD2). In the H_2_O_2_ group, the expression of HIF-1α and HO-1 was significantly increased, while SBT-20 could recover the expression to a normal level. The level of SOD1 and SOD2 were downregulated and partially recovered after SBT-20 treatment in the oxidative stress model (Figure 2B). Higher levels of ROS production were observed in the H_2_O_2_-treated HK-2 cells. Similarly, treatment with the SBT-20 reduced ROS production in the H_2_O_2_-treated HK-2 cells (Figure 2C). The decreased mitochondrial membrane potential was also recovered by SBT-20 treatment in H_2_O_2_-treated HK-2 cells (Figure 2D).

Apoptosis is a process in which cells end their lives according to their own procedures under certain physiological or pathological conditions. A variety of stimulating factors that induce apoptosis to lead to renal dysfunction through the apoptosis of renal tubular epithelial cells, which plays an important role in the pathogenesis of renal failure caused by many causes. Because of the irreversible biological characteristics of necrosis, intervention is carried out according to different targets in the process of apoptosis to inhibit the apoptosis of renal tubular epithelial cells, so as to reduce renal dysfunction or failure. The apoptotic cells could be quantified using flow cytometry. The amounts of apoptotic cells in the SBT-20 treated group decreased more markedly than those H_2_O_2_ treated groups (Figure 2E), which performed the anti-apoptotic ability of SBT-20. The protein level of cleaved-caspase3 was also down-regulated by treating with SBT-20 in H_2_O_2_-treated HK-2 cells (Figure 2F).

### SBT-20 improves renal function in CRF mice

**Figure 3:**
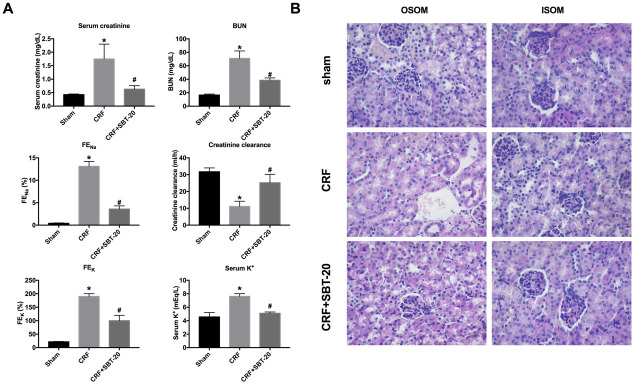
**SBT-20 reduces renal injury in CRF mice.** (**A**) The renal function of mice were evaluated (serum creatinine, BUN, FE_Na_, serum K^+^, FE_K_, and creatinine clearance). n=6. (**B**) Representative image of H&E staining in the outer stripe of the outer medulla (OSOM) and inner stripe of the outer medulla (ISOM). (*P<0.05 *vs.* sham group, ^#^*P*<0.05 *vs.* CRF group).

For further research, the CRF model was constructed on C57BL/6. SBT-20 (5 mg/kg) or saline was injected intraperitoneal to mice after CRF operation. Renal function was determined before sacrificing. CRF mice performed renal function injury by biomarkers results assessment, the up-regulated level of serum creatinine BUN, FE_Na_, serum K^+^, FE_K_, and the down-regulated of, creatinine clearance, which indicated the renal dysfunction. SBT-20 significantly improved all measurements when compared with the CRF group (Figure 3A). In summary, SBT-20 could improve renal dysfunction in CRF mice.

The renal histopathological changes were observed by microscope, and the renal injury was evaluated. The results of pathological sections showed that the renal tissue of CRF mice treated with normal saline had the severe renal tubular injury, and obvious renal tubular necrosis, transparent tubular type, and cell collapse could be seen in the outer stripe of the outer medulla (OSOM) and the inner stripe of the outer medulla (ISOM). Compared with the normal saline group, the renal injury in the SBT-20 treatment group was significantly improved (Figure 3B).

### SBT-20 protect mitochondria protection *in vivo*

**Figure 4:**
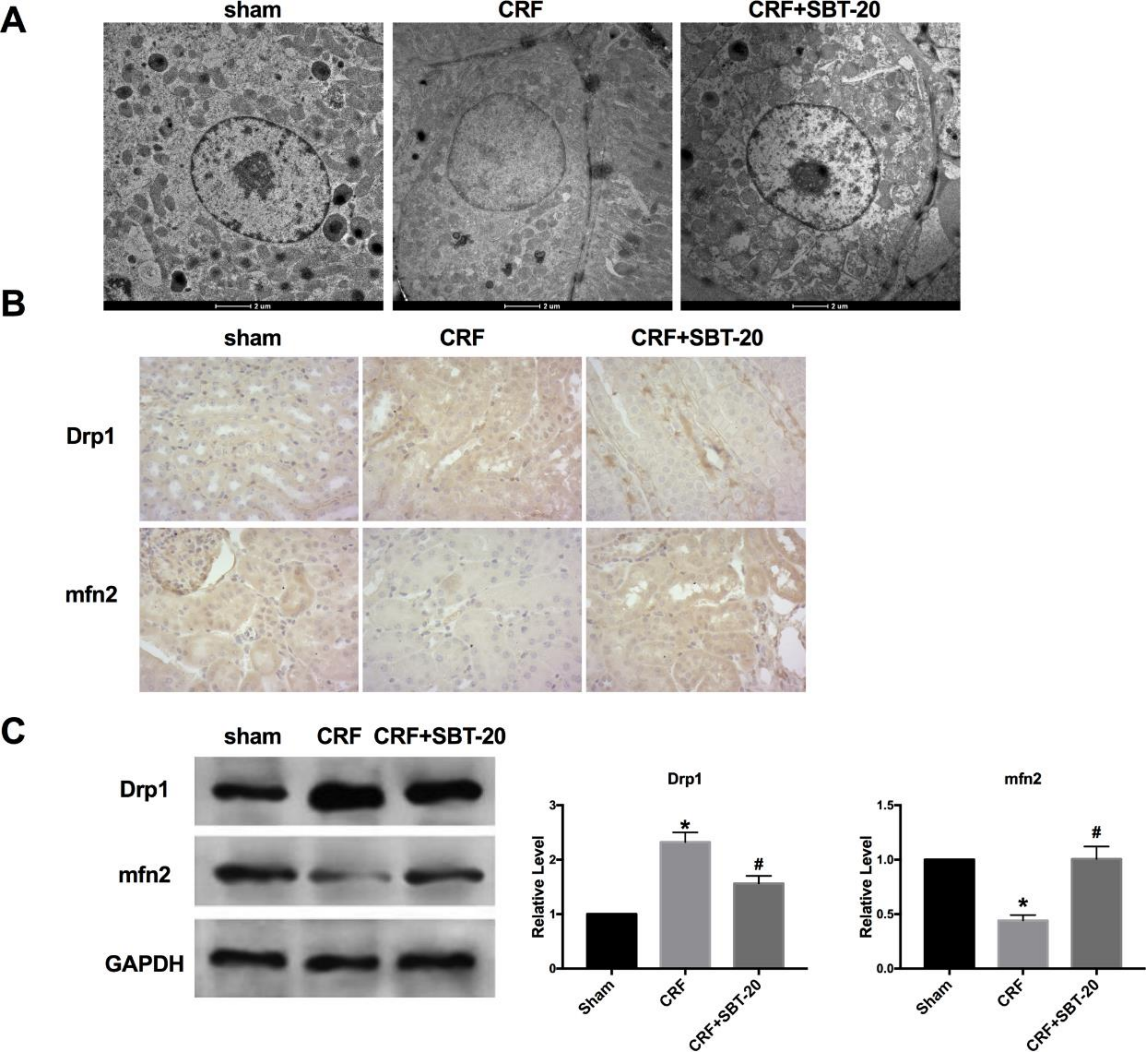
**SBT-20 protect mitochondria protection *in vivo.*** (**A**) Transmission electron microscope image of mitochondria in outer medulla. (**B**) Representative image of Immunohistochemistry (IHC). (**C**) The protein expression of Drp1 and Mfn2 in renal tissues. n=5. (*P<0.05 *vs.* sham group, ^#^*P*<0.05 *vs.* CRF group).

The results of transmission electron microscope showed that the mitochondria of CRF mice were extensively damaged. It is characterized by the loss of round, swollen mitochondrial crest, the destruction of the membrane and the release of matrix material into the cytoplasm. In contrast, SBT-20-treated mice showed many elongated mitochondria, but retained the crest structure in the complete membrane folding at the base of the renal tubular cells. A small amount of swollen mitochondria can be seen in some cells(Figure 4A). Mitochondrial mitotic protein Drp1 regulates mitochondrial metabolism, promotes mitochondrial fragmentation, activates mitochondrial autophagy, regulates mitochondrial outer membrane permeability and participates in the process of apoptosis. Drp1 expression intensity was assessed by performing IHC, and Drp1 level was found to be up-regulated in the CRF mice compared to sham group, and Mfn2 level was decreased in CRF mice compared to sham group. SBT-20 administration dramatically prevented Drp1 expression and recovered Mfn2 expression in CRF mice (Figure 4B). Western blot also showed the similar results, the up-regulated protein expression of Drp1 and down-regulated protein expression of Mfn2 were restored by SBT-20 in renal tissues of CRF mice (Figure 4C).

### SBT-20 prevents inflammation in CRF mice

**Figure 5:**
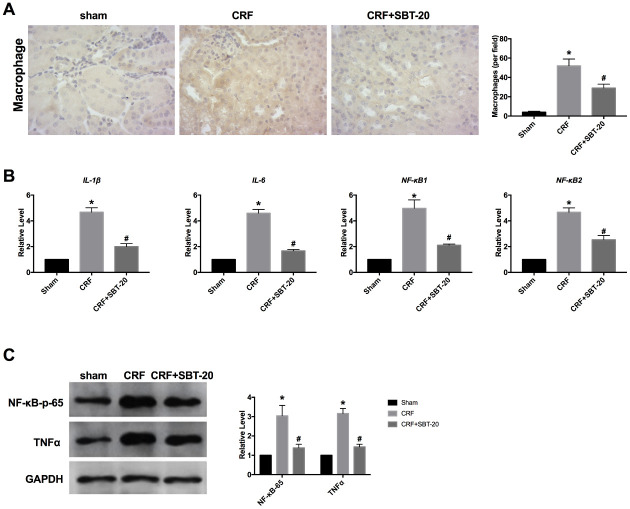
**SBT-20 prevents inflammation in CRF mice.** (**A**) Macrophage infiltration in different group. (**B**) The mRNA level of inflammatory cytokines (IL-1β, IL-6, NF-κB1 and NF-κB2). n=5. (**C**) The protein expression of NF-κB-p65 and TNFα in renal tissues. n=5. (*P<0.05 *vs.* sham group, ^#^*P*<0.05 *vs.* CRF group).

Based on the results *in vitro*, we ensured that SBT-20 could alleviate the inflammation level in LPS-treated HK-2 cells. Further, we explored the anti-inflammation function *in vivo*. Macrophages can release proinflammatory cytokines. Reducing macrophage activation can protect the kidney from inflammation. Macrophage infiltration in SBT-20 group was significantly less than that in normal saline group (Figure 5A). By performing RT-PCR assay, the level of inflammatory cytokines increased in CRF mice, SBT-20 treatment prevent the expression level of the inflammatory-associated cytokine IL-1β, IL-6, NF-κB1 and NF-κB2 in CRF mice (Figure 5B). Western blot also performed the similar results by detecting the level of NF-κB-p65 and TNFα (Figure 5C). In summary, SBT-20 could alleviate inflammation i*n vitro* and * in vivo.*

### SBT-20 induces oxidation decrease in CRF mice

**Figure 6:**
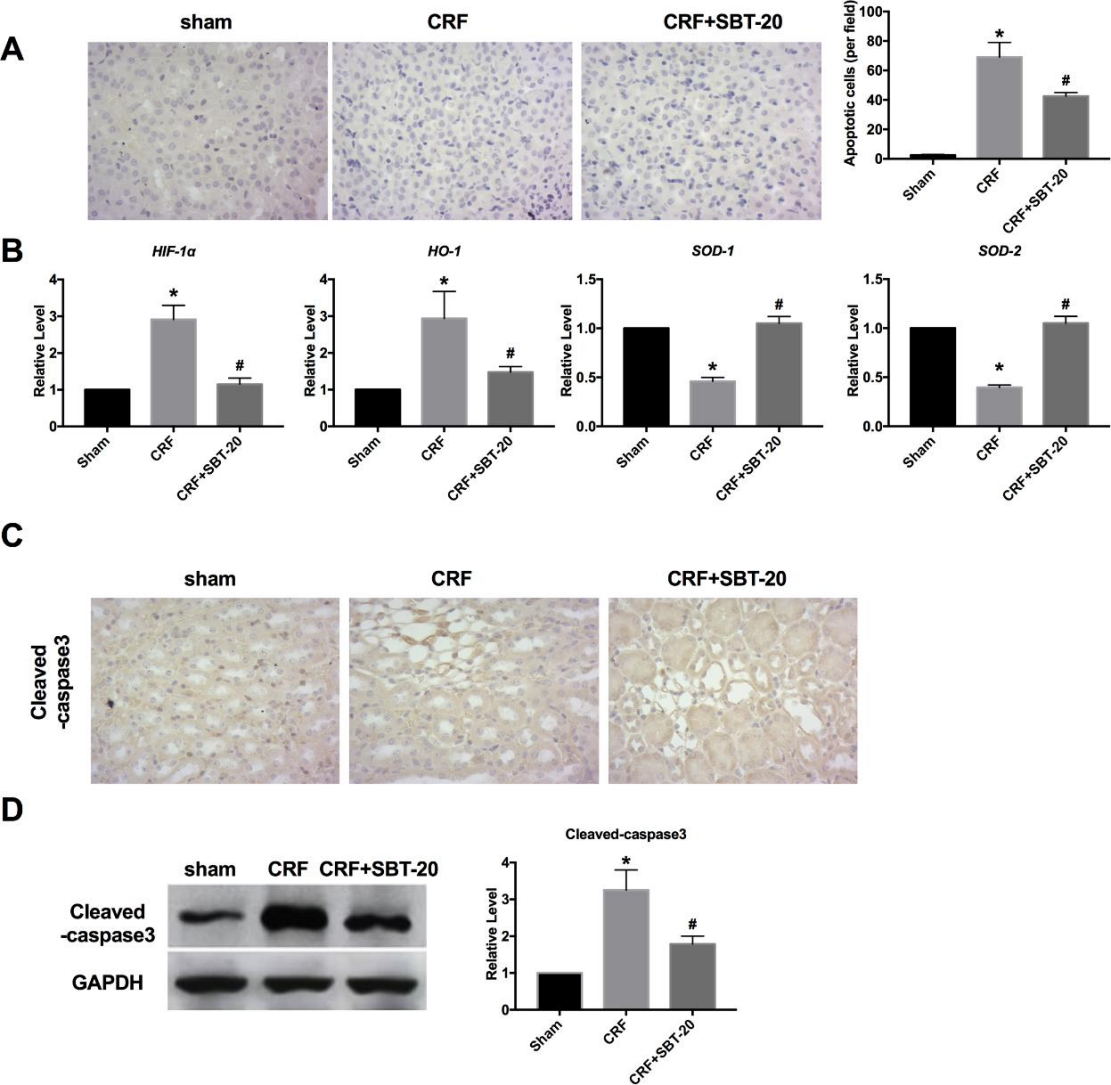
**SBT-20 decreases oxidation in CRF mice.** (**A**) TUNEL assay was performed in mice. (**B**) RT-PCR was performed to detect oxidative stress-relative markers, HIF-1α, HO-1, SOD1 and SOD2. n=5. (**C**) Cleaved-caspase3 expression intensity was evaluated by IHC assay. (**D**) Western blot was performed to measure the expression of cleaved-caspase3 in renal tissues. n=5. (*P<0.05 *vs.* sham group, #*P*<0.05 *vs.* CRF group).

Next, the oxidation stress level was measured *in vivo*. TUNEL detection showed that oxidative stress and apoptosis in renal tubular epithelial cells of CRF mice increased, and SBT-20 administrated significantly decreased the level of apoptosis (Figure 6A). RT-PCR was performed to detect oxidative stress-relative markers, HIF-1α, HO-1, SOD1 and SOD2. In CRF mice group, the expression of HIF-1α and HO-1 were markedly up-regulated, while SBT-20 could block the increased expression of HIF-1α and HO-1. The expression level of SOD1 and SOD2 were downregulated and partially recovered by SBT-20 treatment in CRF mice (Figure 6B). Cleaved-caspase3 expression intensity was evaluated by IHC assay, and cleaved-caspase3 level was found to be up-regulated in the CRF mice. SBT-20 administration dramatically prevented cleaved-caspase3 expression n in CRF mice (Figure 6C). Western blot also showed the similar results, the up-regulated protein expression of cleaved-caspase3 was abolished by SBT-20 in renal tissues of CRF mice (Figure 6D).

## DISCUSSION

At present, the treatment of CRF is mainly symptomatic. The risk factors for correcting the progression of chronic renal failure include avoiding acute worsening factors and reducing the risk factors for the progressive development of chronic renal failure [14], which still need further study.

Mitochondrial dysfunction will lead to inherent renal cell damage and induce cell apoptosis; at the same time, the increase of reactive oxygen species produced by defective mitochondria will aggravate the oxidative stress injury of the kidney [15]. With the occurrence and development of CRF, there are special changes in the morphology and function of mitochondria. Many *in vitro* or animal experiments have found and clarified that renal cell apoptosis is consistent with the degree of glomerulosclerosis under the action of oxygen free radicals [16, 17]. Mitochondrial dysfunction can lead to the decrease of cell energy supply and the increase of oxidative damage, which leads to cell injury and apoptosis and the occurrence and development of glomerular disease [18, 19]. At present, the mechanism of mitochondrial DNA mutation and how mitochondrial damage induces CRF has not been clarified, but many experimental results have confirmed that mtDNA mutation can weaken normal respiratory function and release high levels of ROS, to activate apoptosis and damage to nuclear genome [20]. Here, we assessed the effect of SBT-20 on mitochondrial function. We observed that intracellular uptake of SBT-20 in LPS-stimulated HK-2 cells. Meanwhile, SBT-20 was thought to be unable to clear the nervous system ROS [21]. In this research, the ability of scavenge ROS and mitochondrial function protection was performed in CRF.

A variety of mitochondrial targeting peptides can be used in the treatment of diseases [22, 23]. The new beneficial effect of MitoQ on renal tubular injury in diabetic kidney disease was demonstrated by *in vitro* and *in vivo* models. This process needs to restore the phagocytosis function of renal tubular cells through Nrf2-mediated PINK transcriptional regulation, and improve mitochondrial oxidative stress and abnormal mitochondrial dynamics, resulting in the weakening of renal tubular injury and apoptosis under high glucose conditions [24]. It was found that aldosterone infusion significantly induced renal injury in mice. In addition, Ros production and mitochondrial dysfunction in the kidney of mice perfused with aldosterone were increased, and Nlrp3 inflammatory bodies were activated. We also found that mitochondria-targeted antioxidant treatment has beneficial effects on mitochondrial dysfunction, Nlrp3 inflammatory body activation and renal fibrosis [25]. The mitochondria-targeted peptides has potential application prospect in the treatment of nephropathy.

In previous reports, Toyama S et al. examined the protective effect of SBT-20 against the development of chemotherapy-induced peripheral neuropathy utilizing a murine model of peripheral neuropathy induced by oxaliplatin. This finding suggest that SBT-20 may be a drug candidate for the prevention of chemotherapy-induced peripheral neuropathy [26]. SBT-20 increases efficiency of the electron transport chain and improves coupling of oxidative phosphorylation. SBT-20 treatment after ischemia also significantly reduced interstitial fibrosis. These new findings reveal that enhancing mitochondrial bioenergetics may be an important target for improving ischemia tolerance, and SBT-20 may serve well for minimizing acute kidney injury and chronic kidney disease following surgical procedures such as partial nephrectomy and transplantation [27]. Based on above results, we found that SBT-20 can not only remove ROS products, restore mitochondrial membrane potential and restore mitochondrial function. In histopathological detection, SBT-20 improved severe renal tubular injury, and obvious renal tubular necrosis, transparent tubular type and cell collapse. Further, the accumulated of macrophage also performed the decreased level of inflammation. Meanwhile, SBT-20 alleviated the apoptosis in CRF. Thus it is indicated that SBT-20 is capable of preventing CRF injury.

In recent years, great progress has been made in the study of mitochondrial targeted drug delivery system, but there are still many problems to be solved. After targeting drugs to mitochondria, the transport of drugs in mitochondria is considered to be an important step in the treatment of mitochondrial diseases [28, 29]. For example, apoptosis-inducing drugs and apoptosis inhibitors should be transported to the outer membrane of the mitochondria, because various responses related to apoptosis can be triggered in the outer membrane of the mitochondria, while proteins and coenzymes related to the respiratory chain should be transported to the membrane space and intima of the mitochondria, where there are various electron transport systems related to respiration. Based on this concept, a new concept transport regulation in mitochondria has been proposed to achieve more refined drug delivery. Therefore, the drug effect can be maximized by transporting antioxidants to the gap and intima of mitochondria [22]. With the rapid development of mitochondrial targeting technology, there are many studies on tumor therapy, mitochondrial diseases and central degenerative diseases. Therefore, wit. the development of fine mitochondrial transport regulation technology, mitochondrial targeted antioxidants will become a promising clinical drug.

## CONCLUSIONS

Our results performed that SBT-20 could play an important role in attenuating CRF *in vitro* and *vivo*, possibly by inhibiting ROS production and restoring the mitochondria function, and preventing inflammation and oxidative stress. Therefore, SBT-20 might be an effective strategy disease progression in CRF.

## MATERIALS AND METHODS

### Experimental animal

8 weeks old male C57BL/6 homologous mice were used. The SPF animal room provides constant room temperature, 12 h light/dark cycle, (50 ±5)% humidity, room temperature 23° C. Mouse pellets (20% protein content) and water are supplied by the experimental animal center. Pre-test normal feeding for 1 week, in order to adapt to the environment. The mice were anesthetized with 10% garbital sodium solution (150 μL) intraperitoneally. The mice were fixed on the operating table in the supine position, and the abdomen of the mice was disinfected with 0.5% chlorhexidine solution to disinfect the skin of the operation area. After laparotomy with the left abdominal incision, expose the left kidney, separate the adrenal gland and perirenal fat from the kidney, dissociate the left kidney, and be careful not to accidentally hurt the ureter. After ligation with polyglycolic acid suture, the upper and lower poles of the left kidney (accounting for about 1/3 of the renal volume) were cut off by ophthalmology, and the gelatin sponge was pressed to stop bleeding for 2 min. After resection, the residual kidney was restored to the renal fossa, the organs and peritoneum were covered, and the peritoneum and skin were sutured layer by layer. One week later, the right kidney was exposed by the same method, and the right kidney was resected after ligating the right renal artery and vein and ureter, respectively. After the operation, the mice were put back into the feeding cage after waking up in a warm environment. The operative procedure was about 15 min. In the sham operation group, the kidney was not resected, and the laparotomy and suture were the same as those in the 5/6NX group. Mice were intraperitoneally injected with 3% pentobarbital sodium and were sacrificed by excessive anesthesia with a dose of 90 mL/kg at 6 weeks after the completion of 5 NX operation, and the degree of CRF was measured by blood urea nitrogen (BUN) and serum creatinine (Ser). Intraperitoneal injection of normal saline and SBT-20 (5 mg/kg) [30, 31] was given immediately after CRF operation (Sham, n=10; CRF, n=9; CRF+SBT-20, n=10). The research protocol of this study was approved by the Animal Care and Use Committee of Cangzhou Central Hospital.

### CRF model *in vitro*

HK-2 cells (human tubular epithelial cells) were cultured in plated with normal conditions. HK-2 were treated with LPS (400 ng/mL) /H_2_O_2_ (200 μM) or 12 h. Cells were then treated with SBT-20 (1μM) for 1, 2, 4 and 8 h, and normal cells were also tested as a control. Mitochondria were dyed with Mito-Tracker (250ng/mL) for 0.5 h. After washed with PBS and fixed with 4% formaldehyde buffer, the fluorescence in cells was imaged using confocal microscopy. For investigating intracellular drug release, cells were treated with SBT-20 labeled with FITC following the procedure described above.

### Renal function assessment

Serum and urine samples were analyzed by the lab of Cangzhou Central Hospital. FE_Na (_fractional excretion of sodium) and FE_K_ were calculated as follows: FE_Na_ = (Urinary_Na_ × Serum_creatinine_ /Serum_Na_ × Urinary_creatinine_) × 100% and FE_K_ = (Urinary_K_ × Serum_creatinine_/Serum_K_ × Urinary_creatinine_) × 100%.

### Morphological analysis and immunohistochemistry (IHC)

Morphological of mitochondrial were assessed using transmission electron microscopy (TEM). Paraffin-embedded kidney sections used for IHC studies were dewaxed, rehydrated, and incubated with primary antibodies overnight at 4° C. The sections were subsequently incubated with secondary antibodies, treated with diaminobenzidine, counterstained with hematoxylin, and examined.

### Western blot

Total protein was collected from cells with RIPA lysis Mix (Beyotime, China). Briefly, 60 μg protein extraction was loaded *via* SDS-PAGE and transferred onto nitrocellulose membranes (MILLIPORE, USA), then put them into 5% blocking solution for 2 h. The membranes were incubated with primary antibodies at 4° C for one night. After incubation with secondary antibodies, the membranes were scanned using an Odyssey, and data were analyzed with Odyssey software (LI-COR, USA).

### Real time-PCR

Total RNA was isolated from tissues and cells using Trizol reagent (Invitrogen, Carlsbad, CA, USA) according to the manufacturer’s protocols. Total RNA (1 μg) was used for synthesizing the first-strand cDNA using the cDNA Reverse Transcription Kit (Applied Biosystems, Foster City, Ca, USA). qRT-PCR was performed with the SYBR Green Mix kit (Applied Biosystems) according to the manufacturer’s instructions. The relative RNA levels were calculated using the ΔΔCt method.

### Measurement of ROS production

HK-2 cells were incubated for 30 min with 10 μM 2′,7′- dichlorodihydrofluorescein diacetate (DCF-DA) probe, a fluorogenic dye used for measuring ROS levels within cells. After fixation in 4% paraformaldehyde and PBS washes, cells were incubated in DAPI for 10 min to stain nuclei. Fluorescence signals were analyzed by a fluorescence microscope.

### Mitochondrial membrane potential detection

JC-1 is in a state of aggregation and emitted red fluorescence after staining when the mitochondrial membrane potential is high. Oppositely, JC-1 presents as a monomer and emits green fluorescence after staining when the mitochondrial membrane potential is low. HK-2 cells were cultured with Mitochondrial Membrane Potential Assay Kit with JC-1 (Beyotime) in accordance with the manufacturer’s protocol, and the images were obtained by using the EVOS FL auto cell image system. JC-1 owns a potential-dependent accumulation in mitochondria. Mitochondria with normal membrane potential accumulate and produce red fluorescence when high concentrations of JC-1 are aggravated.

### Immunofluorescence staining

Cells were plated in a 24-well cell culture plate. The cells were washed by PBS and fixed with 4% paraformaldehyde and permeabilized with 0.2% Triton-X-100 solution in PBS. Next, we blocked the cell using goat serum. Then, the cells were incubated with primary antibody at 4° C overnight followed with FITC-conjugated goat anti-mouse antibodies incubation for 1h. After three washes with PBS, we incubated cells by DAPI.

### Cell apoptosis assay

The cells were counted, about 5×10^5^cells/mL. Then, 1 mL cells were centrifuged, 1000 rpm, 10 min, 4°C, and the medium was throw away. The cells were washed with PBS and dropped medium. The cells were resuspended and avoid light for 15 min, 200 μL binding buffer with 10 μL annexin V-FITC, and 10 μL PI. Flow cytometry was used to measure the apoptosis rate within 1 h.

### Statistical analysis

All data are presented as a mean ± S.E.M. We used the unpaired t-test to compare two groups and one-way ANOVA to compare three or more groups. We have considered significant differences when *P* < 0.05.
